# Contribution of red blood cells to the compensation for hypocapnic alkalosis through plasmatic strong ion difference variations

**DOI:** 10.1186/cc9554

**Published:** 2011-03-11

**Authors:** T Langer, L Zani, E Carlesso, A Protti, P Caironi, M Chierichetti, ML Caspani, L Gattinoni

**Affiliations:** 1 Università degli Studi di Milano, Milan, Italy

## Introduction

Chloride shift is the movement of chloride between red blood cells (RBC) and plasma (and *vice versa*) caused by variations in pCO_2_. The aim of our study was to investigate changes in plasmatic strong ion difference (SID) during acute variations in pCO_2 _and their possible role in the compensation for hypocapnic alkalosis.

## Methods

Patients admitted in this year to our ICU requiring extra-corporeal CO_2 _removal were enrolled. Couples of measurements of gases and electrolytes on blood entering (v) and leaving (a) the respiratory membrane were analyzed. SID was calculated as [Na^+^] + [K^+^] + 2[Ca^2+^] - [Cl^-^] - [Lac^-^]. Percentage variations in SID (SID%) were calculated as (SID_v _- SID_a_) × 100/SID_v_. The same calculation was performed for pCO_2 _(pCO_2_%). Comparison between v and a values was performed by paired *t *test or the signed-rank test, as appropriate.

## Results

**Figure 1:**
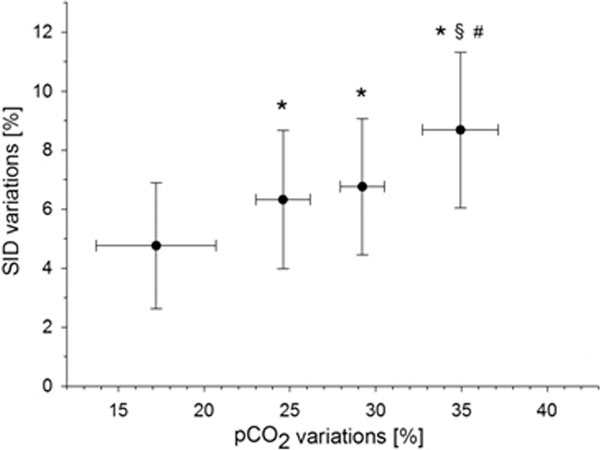
*P < 0.05 versus first quartile. ^§^*P *< 0.05 versus second. ^#^*P *< 0.05 versus third. One-way ANOVA.

Analysis was conducted on 205 sample-couples of six enrolled patients. A significant difference (*P *< 0.001) between mean values of v-a samples was observed for pH (7.41 ± 0.05 vs. 7.51 ± 0.06), pCO_2 _(48 ± 6 vs. 35 ± 7 mmHg), [Na^+^] (136.3 ± 4.0 vs. 135.2 ± 4.0 mEq/l), [Cl^-^] (101.5 ± 5.3 vs. 102.8 ± 5.2 mEq/l) and therefore SID (39.5 ± 4.0 vs. 36.9 ± 4.1 mEq/l). pCO_2_% and SID% significantly correlated (*r*^2 ^= 0.28, *P *< 0.001). Graphical representation by quartiles of pCO_2_% is shown in Figure 1.

## Conclusions

As a reduction in SID decreases pH, the observed movement of anions and cations probably limited the alkalinization caused by hypocapnia. In this model, the only source of electrolytes are blood cells (that is, no interstitium and no influence of the kidney is present); it is therefore conceivable to consider the observed phenomenon as the contribution of RBC for the compensation of acute hypocapnic alkalosis.

